# Pterostilbene suppresses head and neck cancer cell proliferation via induction of apoptosis

**DOI:** 10.55730/1300-0152.2708

**Published:** 2024-08-27

**Authors:** Talih ÖZDAŞ, Sibel ÖZDAŞ, İpek CANATAR, Erdem KAYPAK

**Affiliations:** 1 Department of ENT, Adana City Training and Research Hospital, Health Science University, Adana, Turkiye; 2 Department of Bioengineering, Faculty of Engineering Sciences, Adana Alparslan Türkeş Science and Technology University, Adana, Turkiye

**Keywords:** Head and neck cancer, pterostilbene, anticancer, cytotoxicity, apoptosis

## Abstract

**Background/aim:**

Head and neck cancer (HNC) is one of the most prevalent causes of death worldwide, and so discovering anticancer agents for its treatment is very important. Pterostilbene (PS) is a trans-stilbene reported to be beneficial in managing various cancers. The objective of the study was to evaluate the cytotoxic, antiproliferative, proapoptotic, and antimigrative effect of PS on HEp-2, SCC-90, SCC-9, FaDu, and Detroit-551 cell lines.

**Materials and methods:**

MTT and live/dead assays were employed to assess cell viability, while a cell migration test was performed to evaluate wound healing capacity. The mRNA, protein, and intracellular expression levels of *CASP-3*, *BAX*, and *BCL-2* genes were evaluated by real-time PCR, western blotting, and immunofluorescence staining. Annexin V-PI staining was conducted to assess the amounts of viable, apoptotic, and necrotic cells.

**Results:**

The results revealed that PS displayed cytotoxic, antiproliferative activity in a dose-dependent manner in HNC cells by upregulating *CASP-3* and *BCL-2* while downregulating *BCL-2* in the apoptotic pathway. The proapoptotic properties were confirmed by the annexin-V-IP results. Moreover, PS displayed a significant suppressive efficacy on the migration capacity of HNC cells.

**Conclusion:**

The present study provides proof that PS has the prospective to be improved as an attractive anticancer agent against HNC following advanced studies.

## Graphical Abstract

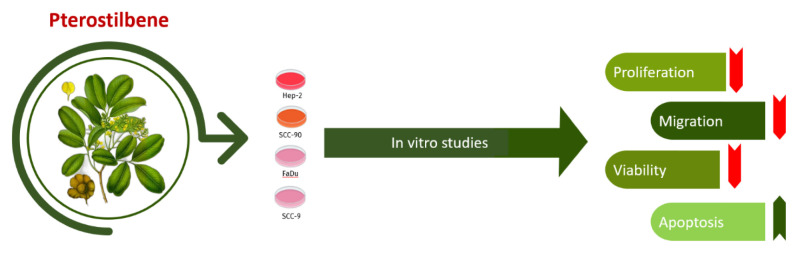

## 1. Introduction

Head and neck cancer (HNC) is one of the most prevalent aggressive cancers globally and has a poor prognosis due to its invasive behavior (Chaturvedi et al., 2011; Barsouk et al., 2023). It impacts about 600,000 patients annually, with a mortality rate ranging from 40% to 50% (Chen et al., 2020). Despite the developments in recent years, the prevalence and mortality of HNC have not decreased sufficiently, improvements in survival rates have not been achieved, and high recurrence and metastasis rates continue to raise doubts about the effectiveness of treatment approaches (Liu et al., 2009; Siegel et al., 2014; Plath et al., 2021). In addition to major clinical interventions such as surgical resection, radiotherapy, and chemotherapy, anticancer agents derived from herbal compounds are increasingly being used for their potential to enhance treatment efficacy in various cancers, including HNC. At the cellular and molecular level, herbal extracts have the potential to influence various malignant indices through specific signaling pathways, offering improved control over HNC malignancy and its clinical complications (Lan et al., 2020).

Ethnobotanical knowledge indicates that many herbal compounds are used in cancer treatment, and studies involving the discovery of many phytochemicals are carried out to investigate the anticancer potential of these compounds (Greenwell and Rahman, 2015). The trans-stilbene compound pterostilbene (PS) is derived from the heartwood of *Pterocarpus marsupium* (Kosuru et al., 2016). PS is an antioxidant and is characterized by low molecular weight and good lipophilicity, allowing it to easily pass the blood–brain barrier (Lin et al., 2009; Ahmad and Kalyanaraman, 2015; Deng et al., 2015). Despite being an analogue of resveratrol, which is widely investigated for its effects on PS, the dimethylether structure of PS enhances lipophilicity and membrane permeability, thereby resulting in higher bioavailability compared to resveratrol (Nagao et al., 2017). Moreover, in vivo animal studies have reported that PS does not have significant toxic effects, making it a promising and potential natural, small molecular drug candidate (Ruiz et al., 2009; Riche et al., 2013). PS has been extensively researched and may offer beneficial effects in preventing and treating cancer, diabetes mellitus, cardiovascular disease, obesity, dyslipidemia, and neurological degeneration (Chen et al., 2018; Ma et al., 2019). Additionally, PS has been proven to suppress cell viability, proliferation, and migration and induce apoptosis in various cancers such as myeloma, leukemia, and gastric, lung, liver, pancreatic, lymphoid, breast, bladder, melanoma, colon, prostate, esophageal, and stomach cancer (Priego et al., 2008; Chen et al., 2018; Lin et al., 2020).

Consequently, PS may reduce the possibility of clinically undesirable side effects, drug resistance, metastasis, and recurrence in the treatment of many cancers such as HNC. The anticancer effects of PS have been associated with mechanisms that activate apoptotic signaling pathways. Therefore, understanding the role of PS in HNC therapy is crucial for developing novel treatment strategies. The aim of the present study was to evaluate the cytotoxic, antiproliferative, proapoptotic, and antimigrative effect of PS against human HNC cell lines through in vitro assays.

## 2. Materials and methods

### 2.1. Cell culture

The majority of HNCs are squamous cell carcinomas, with 27% originating from the larynx, 24% from the oral region, and 6% from the pharynx (Argiris and Eng, 2004). Therefore, HEp-2, SCC-90, SCC-9, and FaDu HNC cancer cell lines were used. Additionally, the Detroit 551 nontumor human fibroblast cell line was used as a control cell line. Cell lines were obtained from the American Type Culture Collection (ATCC). Table 1 shows the cell line characteristics. Throughout the study, it was checked by microscope that the cells had ancestral morphological features. Additionally, the cell lines were evaluated periodically for mycoplasma and other contamination. The cells were cultured in the prepared medium in an incubator with moisturized air at 37 °C containing 5% CO_2_ and observed with an inverted microscope until they became confluent (Ma et al., 2011).

### 2.2. MTT assay

The effectiveness of PS on cell viability was assessed using the MTT assay. The cells were cultivated in an incubator overnight, then the cell medium was removed, and PS (5, 10, 25, 50, 75, and 100 μM) was applied to the cells. The MTT assay was performed at the end of 24 and 48 h. The absorbance (Ab) was measured with a spectrophotometer at 570 nm wavelength. The concentrations at which PS inhibited the cell viability by 10%, 30%, and 50% (IC_10_, IC_30_, and IC_50_) were determined. Cell viability was calculated as a percentage with the following formula (Chang et al., 2018):


$$\text{Cell viability }(\%)=(\text{average }{\text{Ab}}_{570}\;\text{of the test})/(\text{average }{\text{Ab}}_{570}\;\text{of the control})\times 100.$$

### 2.3. Live/dead assay

The effect of PS on cell viability was evaluated through live/dead staining. The cells were cultured for up to 48 h, the medium was eliminated, and the cells were subjected to various doses of PS. The cells were incubated with Eth-1 and C-AM solutions in the dark at room temperature for up to 1 h. The live and dead cells were imaged under a microscope. The viability of cells was assessed by calculating the percentage of viable cells in comparison with the total cell number (Derici et al., 2021).

### 2.4. Cell migration test

The effect of PS doses on the migration capacity of cells was evaluated (Ma et al., 2011). The cells were cultured for up to 24 h. Following this, the medium was removed and a straight scratch was created in each well. After being washed with PBS, the cells were exposed to doses of PS. Then the wound area was imaged with microscope at 0, 24, 36, 48, and 72 h. The scratch wound area was determined using the following formula in ImageJ software:


Scratch wound closure rate (%)=[(Initial scratch area width-Final scratch area width)/(Initial scratch area width)]×10

### 2.5. Real-time PCR

The mRNA expression levels of *CASP-3*, *BAX*, and *BCL-2* genes related to the apoptotic pathway was determined by real-time PCR (Özdaş et al., 2020). TRIzol reagent was utilized for the extraction of total RNA. The RNA was converted into cDNA form with a commercial kit. SYBR green PCR Master Mix was prepared to a final volume of 25 μL for each sample, and a real-time PCR study was performed using 45 cycles at the appropriate annealing temperature for each primer set on a Rotor Gene Q device (Rørth, 2012; Özdaş et al., 2020). For normalization, *GAPDH* was used. The results were analyzed by the comparative cycle threshold (CT) method.

### 2.6. Western blotting

The protein expression level of CASP-3, BAX, and BCL-2 was evaluated by western blotting (Mena et al., 2012). For total protein isolation, RIPA buffer and protease inhibitor were used. Protein samples were equalized to 20 μg, loaded onto SDS-PAGE (10%), and separated by running at 100 V for 90 min in buffer. The proteins from the gel were transferred to the membrane. Blocking of the membranes was performed using 5% skim milk and then treated with primary anti-Casp-3 antibody, anti-Bax antibody, anti-Bcl-2 antibody (1:500, v/v), and anti-β-actin-antibody (1:100, v/v). Then the secondary antibodies at room temperature were washed and imaged with chemiluminescent solution. Protein amount was determined densitometrically using ImageLab version 6.1 software (Mena et al., 2012; Chang et al., 2018).

### 2.7. Immunofluorescence staining method

To evaluate the intracellular expression of CASP-3, BAX, and BCL-2, immunofluorescence staining was performed. After cells were fixed with 4% paraformaldehyde, they were permeabilized using 0.1% (v/v) Triton X-100 and blocked with 1% (v/v) BSA. Afterward, they were treated with anti-Casp-3 antibody (1:1000, v/v) and anti-Bax antibody and anti-Bcl-2 antibody (1:500, v/v) as primary antibodies. Then they were treated with secondary antibodies. Further, DAPI was employed to stain the cell nuclei. The fluorescence signal intensity was assessed using ImageJ.

### 2.8. Annexin V-propidium iodide (PI) staining

In order to evaluate early/late apoptosis in cells, annexin V-PI staining was performed. Cells were treated with PS for up to 48 h and stained with annexin V-PI. The cells were then incubated in the dark, and each sample was measured with a laser at 488 nm in a flow cytometer (Crowley et al., 2016).

### 2.9. Statistical analysis

Statistical analysis of the data was performed using GraphPad Prism version 8.4.3 (Graphpad Software Inc., USA). Every method was replicated a minimum of three times. The data were presented as the mean ± standard deviation. The IC values of PS were analyzed by nonlinear regression model. One-way ANOVA with the post-hoc Tukey test were used to compare the data. A p-value <0.05 was considered statistically significant.

## 3. Results

### 3.1. The antiproliferative and cytotoxic effect of pterostilbene on HNC cells

**Figure 1:**
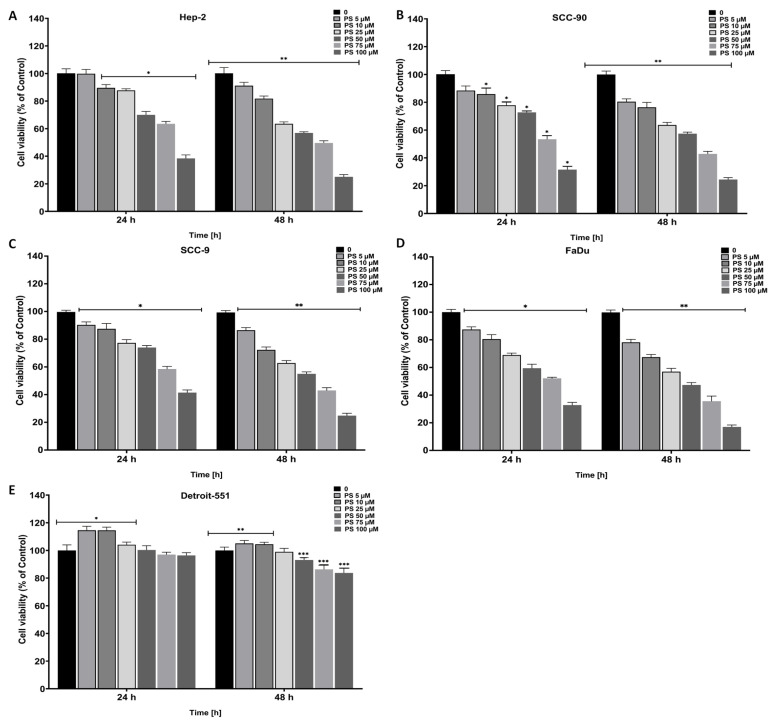
Effect of pterostilbene on the viability of head and neck cancer cells. Cells were treated with increasing concentrations of PS for 24 and 48 h. MTT assay data for PS on **A**. HEp-2, **B** SCC-90, **C**. SCC-9, **D**. FaDu, and **E**. Detroit-551 cells are shown. **p < 0.005, **p < 0.05 for 24 and 48 h are significantly different from untreated head and neck cancer cells. PS: Pterostilbene; MTT: 3-(4,5-Dimethylthiazol-2-yl)-2,5-diphenyltetrazolium bromide.

**Figure 2:**
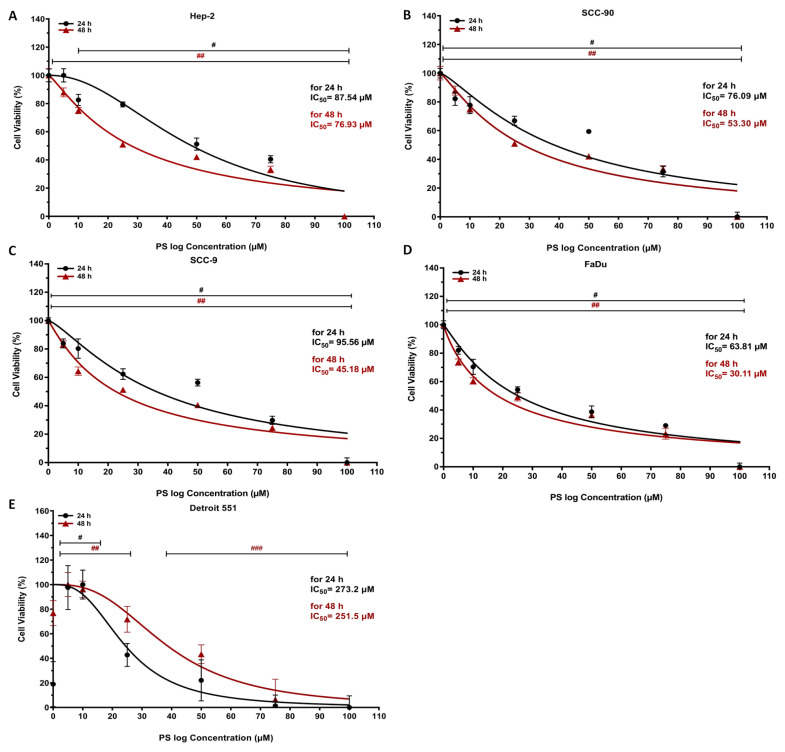
Quantitative evaluation of pterostilbene on cell viability. **A**. HEp-2, **B**. SCC-90. **C**. SCC-9, **D**. FaDu, and **E**. Detroit-551 cells treated with PS for 24 and 48 h were calculated using a nonlinear regression model. ^≠^p < 0.05, ^≠≠^p < 0.05 for 24 and 48 h are significantly different from untreated head and neck cancer cells. PS: Pterostilbene; IC: Maximum inhibition concentration.

HNC cells were treated with certain concentrations (5, 10, 25, 50, 75, and 100 μM) of PS for 24 and 48 h and the proliferation was investigated by MTT assay (Figure 1). The results revealed that PS doses less than 10 μM for Hep-2, SCC-9, and FaDu cells and 5 μM for SSC-90 cells did not display any significant effect on the cell proliferation until 24 h (p < 0.05 for ≥10 μM in Hep-2; p < 0.05 for all doses in SCC-90, SCC-9, and FaDu). However, >5 μM of PS showed an important antiproliferation effect on all HNC cells up to 48 h (p < 0.05 for all). Moreover, at a higher concentration of PS (100 μM), cell proliferation was considerably reduced at the end of 24 and 48 h of incubation. The MTT results showed that treatment of PS in HEp-2, SCC-90, SCC-9, and FaDu cells notably suppressed cell survival in a time- and concentration-dependent manner. Additionally, at the end of 24 h of incubation, PS displayed significant cytotoxicity against the Hep-2, SCC-90, SCC-9 and FaDu cells, particularly at higher concentrations (>50 μM), with IC_50_ values of 87.54 ± 1.94 μM, 76.09 ± 1.88 μM, 95.56 ± 3.78 μM, and 63.81 ± 2.96 μM, respectively. Moreover, at the end of 48 h of incubation, PS displayed significant cytotoxicity against the Hep-2, SCC-90, SCC-9 and FaDu cells, particularly at higher concentrations (>25 μM), with IC_50_ values of 76.93 ± 1.72 μM, 53.30 ± 1.72 μM, 45.18 ± 04.12 μM, and 30.11 ± 3.13 μM, respectively. However, PS had much less cytotoxic effect on nontumor human fibroblast Detroit-551 cells at higher concentrations (>100 μM), with IC_50_ values of 273.2 ± 2.436 μM and 251.5 ± 2.40 μM after 24 and 48 h of incubation, respectively (p < 0.05 for ≥50 μM at 24 h and 48 h) (Figure 2). According to the selectivity index (SI), PS has high selectivity (in the range 2.5–4.48 and 3.26–8.35 for 24 and 48 h) against HNC cells compared to normal cells (Table 2). The results clearly show that PS caused a reduction in HNC cell proliferation that depended on both time and concentration.

### 3.2. The effect of pterostilbene on the viability of HNC cells using the live/dead assay

**Figure 3:**
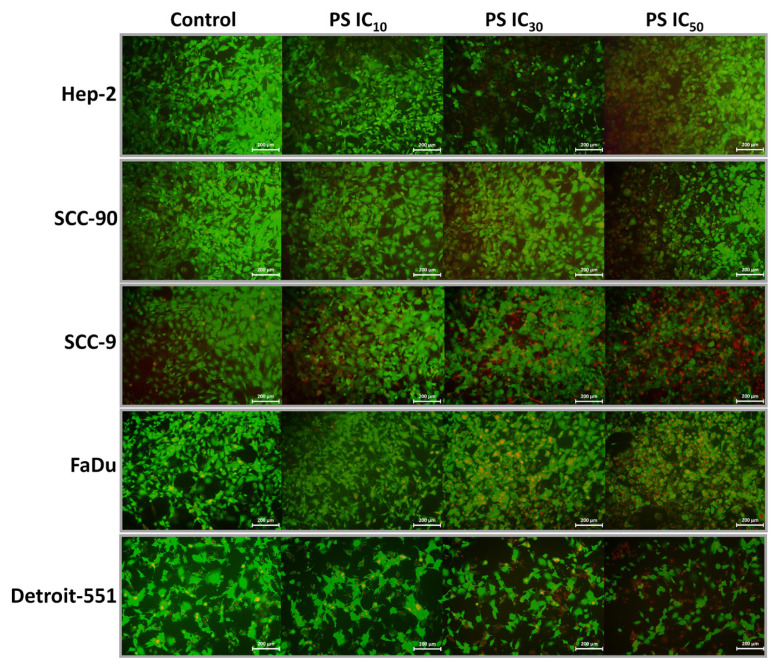
Live/dead cell assay data showing the effects of pterostilbene on the viability of head and neck cancer cells. Representative microscopic images at 10× magnification show C-AM and Eth-1 staining of PS-treated and untreated (scale bar: 200 μm). **A**. HEp-2, **B**. SCC-90, **C**. SCC-9, **D**. FaDu, and **E**. Detroit-551 cells treated with PS at IC_10_, IC_30_, and IC_50_ doses for 48 h were stained with the C-AM (green) and Eth-1 (red). PS: Pterostilbene; Eth-1: Ethidium homodimer-1; C-AM: Calcein-Acetomethoxy.

**Figure 4:**
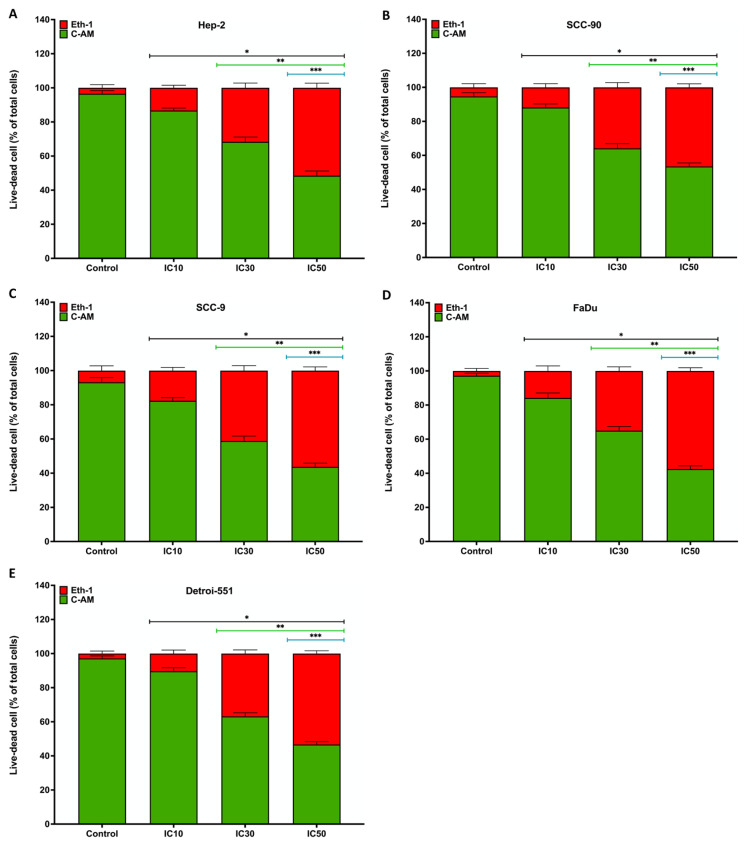
Quantitative assessment of the live/dead cell assay showing the effects of pterostilbene on the viability of head and neck cancer cells. **A**. HEp-2, **B**. SCC-90, **C**. SCC-9, **D**. FaDu, and **E**. Detroit-551 cells show live/dead assay analysis. Cell viability was calculated using the percentage of viable cells compared to the total cell number. Statistical significance is denoted as follows: *p < 0.05, **p < 0.05, and ***p < 0.0001 compared to the control, IC_10_, and IC_30_, respectively.

The cytotoxic effect of PS on HNC cell lines was confirmed 48 h later using a live/dead assay. CAM (green) fluorescence intensity exhibited a concentration-dependent decrease, while Eth-1 (red) fluorescence showed an increase in HNC cells in the treatment group compared to controls under an inverted microscope (Figure 3). According to the quantitative data, the toxic effect of PS on cell viability was concentration dependent in Hep-2 (10.18%, 29.18%, and 49.74%) SCC-90 (6.89%, 32.21%, and 43.48%), SCC-9 (11.62%, 36.85%, and 53.13%), FaDu (13.37%, 33.10%, and 56.26%), and Detroit-551 cells treated with IC_10_, IC_30_, and IC_50_ compared with the control cells (p < 0.05 for all) (Figure 4).

### 3.3. The antimigratory effect of pterostilbene on HNC cells

**Figure 5:**
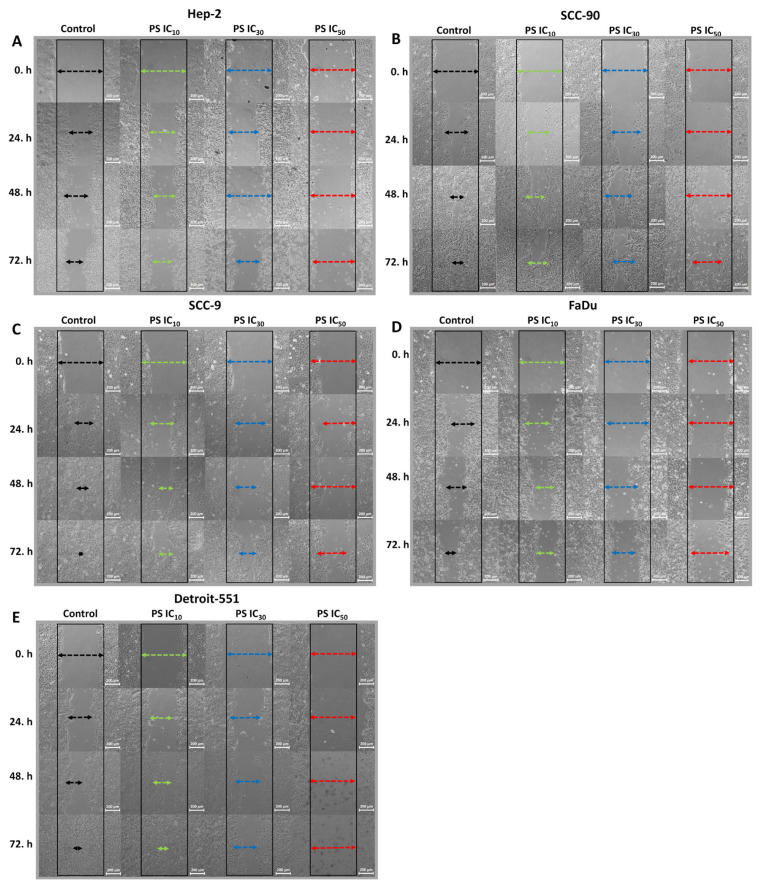
The scratch assay showed the effect of pterostilbene on head and neck cancer cell mobility. Untreated cells cultured in 6-well plates were used as control. The scratch assay was utilized to determine the cell migration potential in cells treated with PS at IC_10_, IC_30_, and IC_50_ concentrations. Representative microscopic images (10× magnification) obtained from **A**. HEp-2, **B**. SCC-90, **C**. SCC-9, **D**. FaDu, and **E** Detroit-551 cells at 0, 24, 48, and 72 h illustrate the wound healing effects of PS (scale bar: 100 μm). The black (initial wound boundary) and colored (final wound boundary) lines depict the edges of the wound area within cell groups.

**Figure 6:**
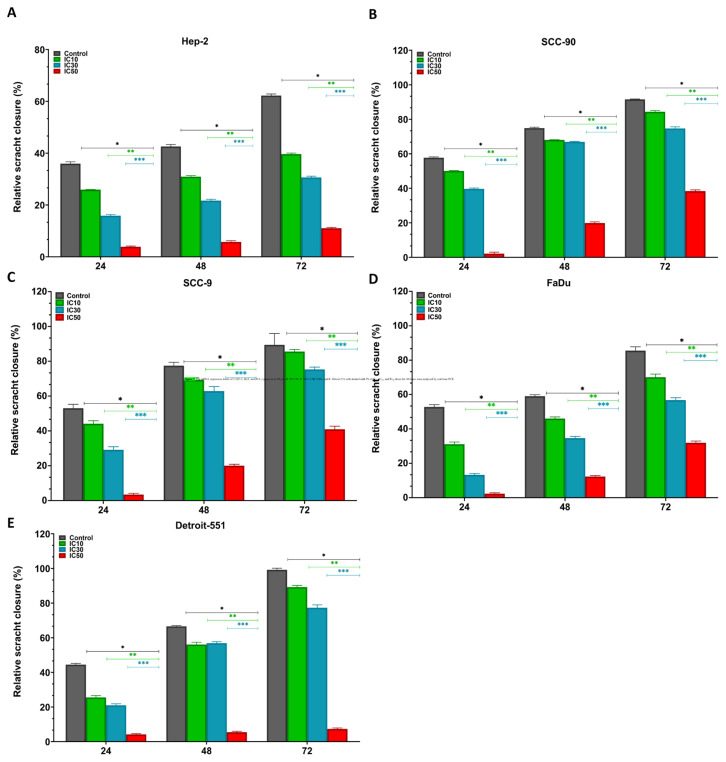
Quantitative assessment of the scratch assay showing the effects of pterostilbene on the mobility of head and neck cancer cells. **A**. HEp-2, **B**. SCC-90. **C**. SCC-9, **E**. FaDu, and **F**. Detroit-551 cells show scratch wound area closure analysis. The wound closure rate of the scratch was measured using ImageJ software. *p < 0.05, **p < 0.05, and ***p < 0.05 compared to the control, IC_10_, and IC_30_, respectively.

The impact of PS on the cell migration capability of HNC lines was assessed by cell migration test. According to the scratch wound closure results, HEp-2, SCC-90, SCC-9, and FaDu cancer cells treated with IC_10_, IC_30_, and IC_50_ doses of PS exhibited significantly suppressed cell mobility with a notably larger wound area, especially after 24 h compared to the control cells (Figure 5). Moreover, the scratch wound space in HNC cell lines treated with PS was noticeably more expansive than those in the control cells, in a concentration-dependent manner at all time points. In addition, the migration activity pattern of IC doses of PS on HNC cell lines was similar up to 72 h (p < 0.0001 for all time points and PS concentrations). The data show that PS has the potential to effectively suppress HNC metastasis (Figure 6).

### 3.4. Effect of pterostilbene on the mRNA, protein, and intracellular expression levels of *CASP-3*, *BAX*, and *BCL-2* genes in HNC cells by real-time PCR, western blotting, and immunofluorescence staining

**Figure 7:**
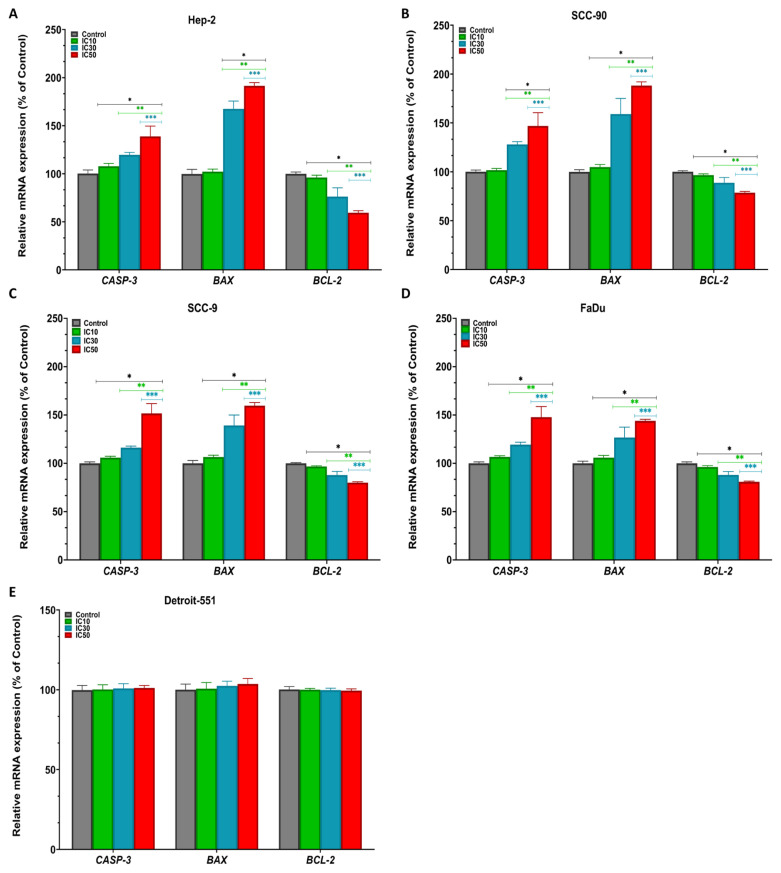
The mRNA expression levels of *CASP-3*, *BAX*, and *BCL-2* genes in **A**. HEp-2, **B**. SCC-90, **C**. SCC-9, **D**. FaDu, and **E**. Detroit-551 cells treated with pterostilbene at IC_10_, IC_30_, and IC_50_ doses for 48 h were analyzed by real-time PCR. The *GAPDH* gene was used for normalization. *p < 0.05, **p < 0.05, and ***p < 0.05 compared to the control, IC_10_, and IC_30_, respectively. *GAPDH*: Glyceraldehyde-3-phosphate dehydrogenase; *CASP-3*: Caspase-3; *BAX*: Bcl-2-associated X; *BCL-2*: B-cell lymphoma 2; CT: Cycle threshold.

**Figure 8:**
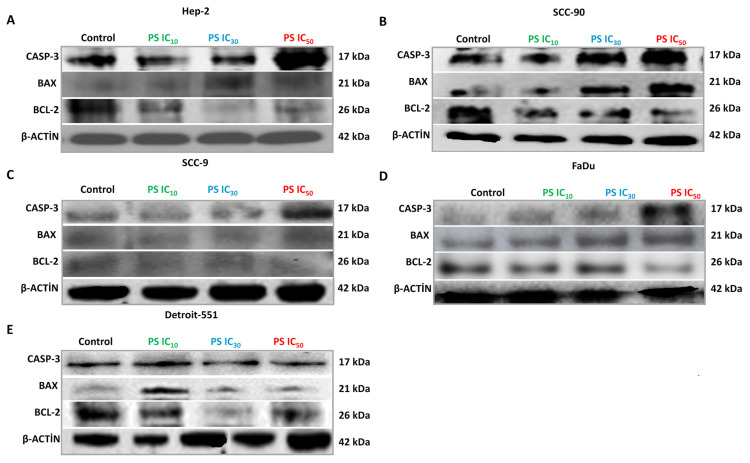
Western blot data showing the effect of pterostilbene on CASP-3, BAX, and BCL-2 protein expression levels in head and neck cancer cells. The protein expression levels of CASP-3, BAX, and BCL-2 were analyzed by western blotting in cells treated with PS at IC_10_, IC_30_, and IC_50_ doses for 48 h.

**Figure 9:**
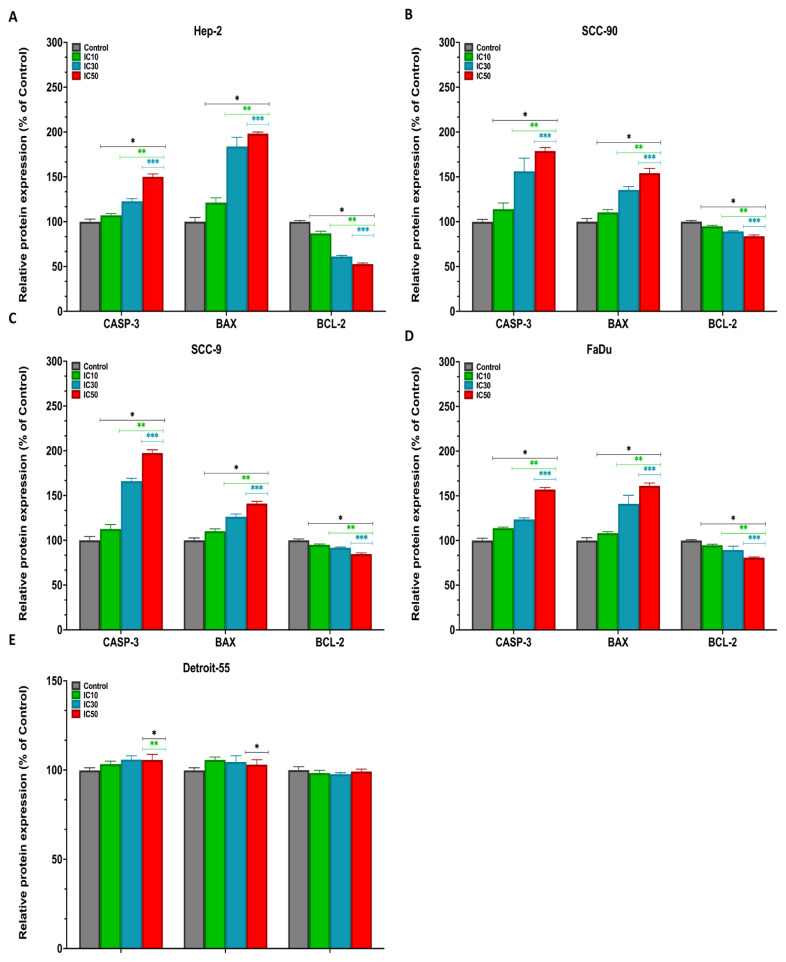
Densitometric analysis of the relative intensity of protein bands obtained from head and neck cancer cells. β-actin (42 kDa) was used for normalization. Data were analyzed using ImageLab software. *p < 0.05, **p < 0.05, and ***p < 0.05 compared to the control, IC_10_, and IC_30_, respectively. The relative expression levels of CASP-3 (17 kDa), BAX (21 kDa), and BCL-2 (26 kDa) in **A**. HEp-2, **B**. SCC-90 and **C**. SCC-9, **D**. FaDu, and **E**. Detroit-551 cells.

To further elucidate the mechanism of the cytotoxic and antiproliferative effects of PS in HNC cell lines, we evaluated the mRNA and protein expression levels of proapoptotic and antiapoptotic genes using real-time PCR and western blotting (Rørth, 2012; Özdaş et al., 2020). The mRNA and protein expression profiles of *CASP-3*, *BAX*, and *BCL-2* were compared after 48 h of treatment with IC_10_, IC_30_, and IC_50_ doses of PS. According to the real-time PCR and densitometric analysis results, PS in HEp-2, SCC-90, SCC-9, and FaDu cells displayed a significant enhancement in a concentration-dependent manner in mRNA and protein expression levels of *CASP-3* and *BAX* genes in comparison with those of the control cells (p < 0.05 for all) (Figure 7). However, treatment of PS resulted in repression of the expression of *BCL-2* (p < 0.05 for all). Further, in the western blot, CASP-3 and BAX protein bands were clearly visible in PS-treated HNC cells, compared to the control cells, while BCL-2 protein bands were faint (Figures 8 and 9).

**Figure 10:**
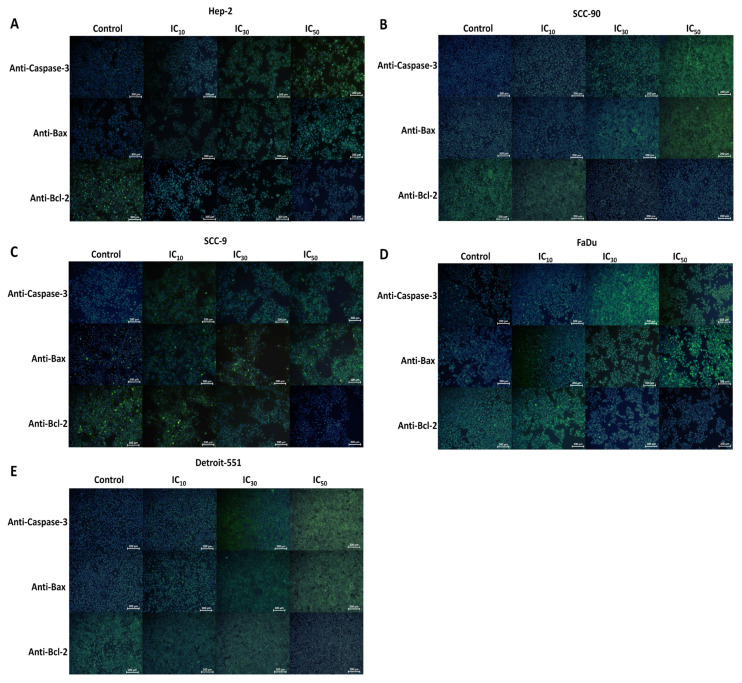
Immunofluorescence staining data demonstrating the effect of pterostilbene on the intracellular expression levels of CASP-3, BAX, and BCL-2 in head and neck cancer cells. Cells treated with PS at IC_10_, IC_30_, and IC_50_ doses for 48 h were analyzed for CASP-3, BAX, and BCL-2 intracellular expression levels through immunofluorescence staining. Immunofluorescence images depicting staining for **A**. HEp-2, **B**. SCC-90, **C**. SCC-9, **D**. FaDu, and **E**. Detroit-551 cells (10×; scale bar: 200 μm).

**Figure 11:**
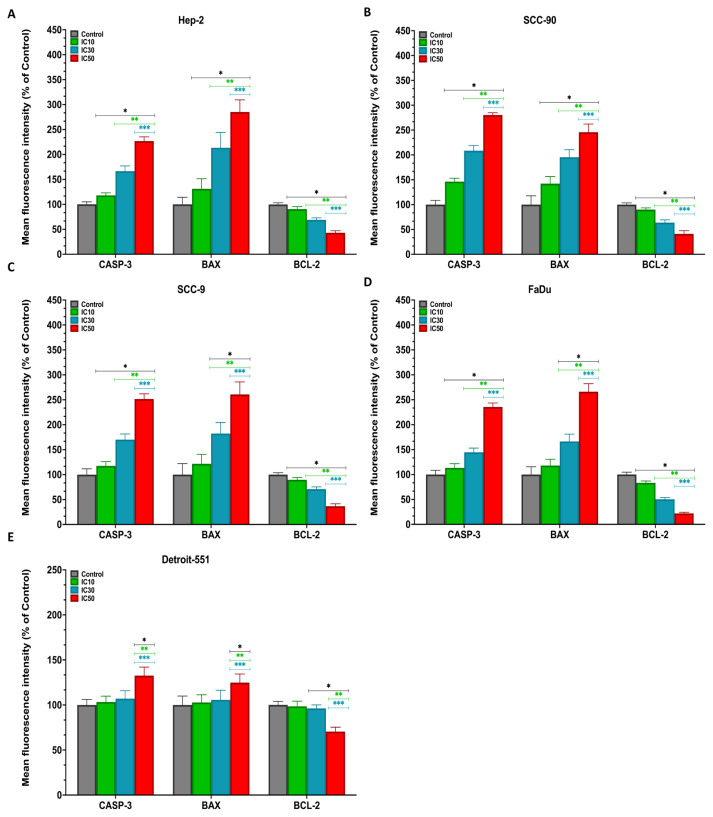
Quantification data for the expression levels of anti-Casp-3, anti-Bax, and anti-Bcl-2 antibodies for **A**. HEp-2, **B**. SCC-90, **C**. SCC-9, **D**. FaDu, and **E**. Detroit-551 cells. The analysis of the relative density of anti-Casp-3, anti-Bax, and anti-Bcl-2 antibodies obtained from head and neck cancer cells using ImageJ software. *p < 0.05, **p < 0.05, and ***p < 0.05 compared to the control, IC_10_, and IC_30_, respectively.

The immunofluorescence staining microscopic images revealed an increase in the fluorescence intensity of anti-Casp-3 and anti-Bax antibodies, but a decrease in anti-Bcl-2 antibody in HEp-2, SCC-90, SCC-9, and FaDu cells treated with IC doses of PS (Figure 10). Moreover, the immunofluorescence staining analysis results showed that treatment with PS induced an elevation in the expression levels of CASP-3 and BAX, while concurrently reducing the expression of BCL-2 in HNC cells compared with the controls (p < 0.05 for all). The expression profile results show that PS affects intrinsic apoptotic-signal pathways by upregulating CASP-3 and BAX and downregulating BCL-2 (Figure 11).

### 3.5. Effect of pterostilbene on apoptosis in HNC cells by annexin V-PI staining

**Figure 12:**
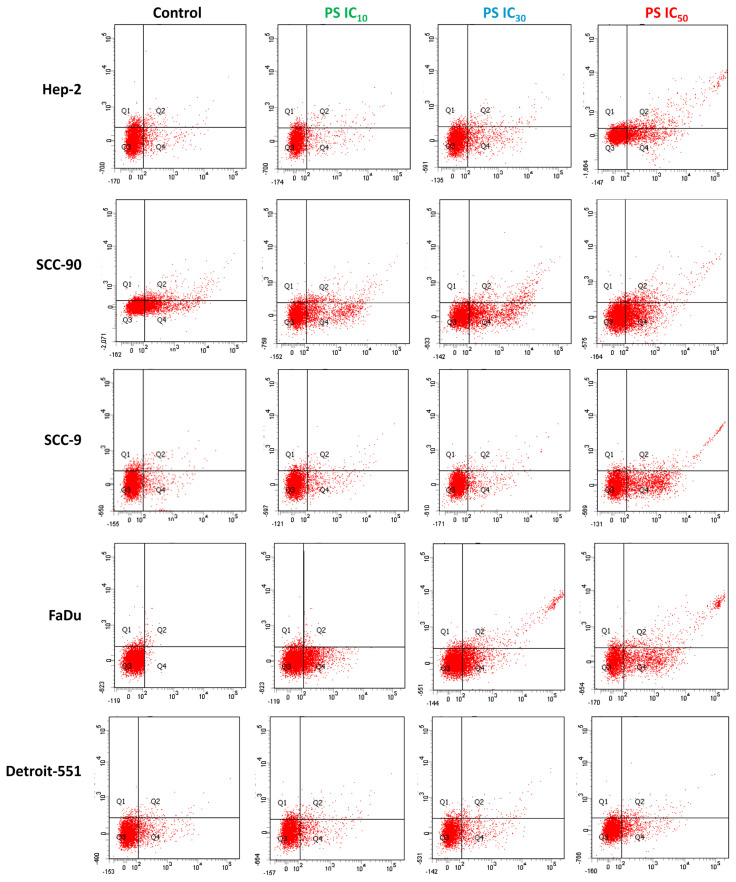
Flow cytometry data of annexin V-PI staining showing the impact of pterostilbene on apoptosis and necrosis in head and neck cancer cells. Cells cultured in a 6-well plate and treated with PS at IC_10_, IC_30_, and IC_50_ doses for 48 h. After staining with annexin V and PI, cells were assessed for apoptosis using flow cytometry. Representative flow cytometry images from **A**. HEp-2, **B**. SCC-90, **C**. SCC-9, **D**. FaDu, and **E**. Detroit-551 cells illustrate the levels of annexin V and PI staining detected in the cells. Q1 denotes necrotic/dead cells in the upper left area, Q2 represents late apoptosis in the upper right area, Q3 denotes live cells in the lower left area, and Q4 indicates early apoptosis in the lower right area. PI: Propidium iodide.

**Figure 13:**
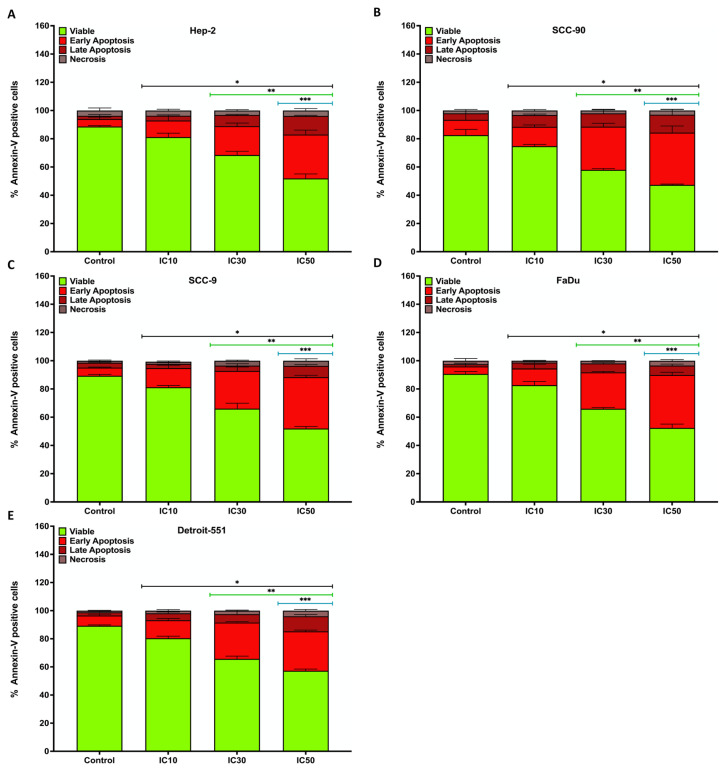
The analysis of the measurement of annexin V and PI staining levels detected in head and neck cancer cells by flow cytometry. Quantification data for the levels of annexin V and PI staining for **A**. HEp-2, **B**. SCC-90, **C**. SCC-9, **D**. FaDu, and **E**. Detroit-551 cells. *p < 0.05, **p < 0.05, and ***p < 0.05 compared to the control, IC_10_, and IC_30_, respectively.

To understand how PS exhibits an antiproliferative effect on HNC cells, annexin V-PI staining was conducted and analyzed with flow cytometry (Crowley et al., 2016). The staining revealed that apoptosis in HEp-2, SCC-90, SCC-9, and FaDu cells was responsible for suppressing cell proliferation by PS (Figure 12). In addition, flow cytometry of Hep-2 (98.24%, 273.24%, and 482.45%), SCC-90 (42.98%, 158.96%, and 221.38%), SCC-9 (79.34%, 230.79%, and 381.88% ± 15.56), and FaDu (8.89%, 27.25%, and 42.21%) cells treated with IC_10_, IC_30_, and IC_50_ doses of PS compared with the controls revealed a remarkable increase in early/late apoptotic cell death in a concentration-dependent manner (p < 0.05 for all) (Figure 13).

## 4. Discussion

Recent pharmacological studies have reported that PS suppresses cell proliferation through the induction of apoptosis by targeting various molecular and signaling pathways (Priego et al., 2008; Chen et al., 2018; Liu et al., 2020). Therefore, in the present study, we demonstrated in vitro the cytotoxic, antiproliferative, proapoptotic, and antimigratory effects of PS on HNC cell lines.

The fundamental feature of cancer is unlimited cell proliferation, and the loss of cell cycle regulation is a critical factor. Therefore, cell proliferation is a significant parameter to be monitored in antitumor therapies (Sherr and Bartek, 2017). In many in vitro and in vivo studies, PS has been shown to suppress cancer cell proliferation through multiple pathways (Li et al., 2013). In studies performed in vitro the treatment of endometrial (37.5–300 μM for ECC-1 and ER/PR), breast (40–80 μM for MCF-7), prostate cancer (40–80 μM for PC3), myeloma (10–50 μM for MM), and lymphoma (12.5–100 μM for DLBCL) cancer cell lines with PS in different concentrations led to a concentration-dependent inhibition of cell viability and proliferation (Chakraborty et al., 2010; Kong et al., 2016; Xie et al., 2016; Wen et al., 2017). This effect was associated with a decrease in mitochondrial membrane potential, elevation in the production of reactive oxygen species (ROS), and activation of caspases (Kong et al., 2016). In their study, according to the MTT data, treatment of PS (5–100 μM) in HEp-2, SCC-90, SCC-9, and FaDu exhibited significant inhibition of cell proliferation in a time- and concentration-dependent manner with high selectivity compared to normal healthy cells, and the results of the live/dead cell assay were consistent with these data. However, our results demonstrated that PS had much less effect on nontumor human fibroblast Detroit-551 cells. Our findings align with those in the literature, indicating the inhibitory effect of PS on cancer cell proliferation.

The doses of PS required to inhibit cell viability and growth may vary according to the type of cancer cell. For example, the IC_50_ values of PS in the breast (fMCF-7), bladder (T24, T24R), endometrial (HEC-1A and ECC-1), and prostate cancer (PC3) cells were calculated to be 65.6 μM, 66.58 μM, 77.95 μM, 72 μM, 78 μM, and 74.3 μM, respectively (Chakraborty et al., 2010; Wen et al., 2017). Additionally, the IC_50_ value of PS in the breast (MDA-MB-231), colon cancer (COLO-205 and HT-29), gastric adenocarcinoma (AGS), leukemia (HL-60), hepatocellular carcinoma (HepG2), colorectal (HCT-116), melanoma (A-375), and prostate (PC-3) cancer cells was 40.6 μM, 71.2 μM, 71.8 μM, 50.7 μM, 46.7 μM, 45.3 μM, 16–82.8 μM, 3.9 μM, and 421 μM, respectively, and no cytotoxic effect was observed in PMNs or HUVEC cells (Remsberg et al., 2008; Ma et al., 2019). In our study, the SCC-9 (IC_50_ 45.18–95.56 μM) and FaDu (IC_50_ 30.11–63.81 μM) cell lines were more sensitive to PS treatment than the HEp-2 (IC_50_ 76.93–87.54 μM) and SCC-90 (IC_50_ 53.30–76.09 μM) cell lines. However, PS did not show cytotoxic effects on the Detroit-551 fibroblast cells. This trend of variability in sensitivity may be associated with different cell lines and their protein expression profiles.

Targeting cell mobility, invasion, and migration in cancer cells is an important strategy for cancer therapies (Friedl and Wolf, 2003). PS treatment in human ovarian (18.5–300 μM for OVCAR-8, Caov-3) and cervical (20–40 μM for HeLa) cancer cells caused apoptosis induction, decreased cell proliferation, and migration through various signaling pathways (Wen et al., 2017; Shin et al., 2020). Previous studies reported that PS caused a concentration-dependent delay in cell migration in bile duct (GBC-SD and SGC-996) and breast (Hs578t and MDA-MB-231) cancer cells compared to controls (Su et al., 2015; Tong et al., 2021). Additionally, hepatocyte, breast, and prostate cancer animal model studies reported that PS treatment significantly suppressed cell invasion, migration, and metastasis (Li et al., 2013; Su et al., 2015). These findings support the evidence that PS inhibits cell migration and invasion potential through upregulation of E-cadherin and downregulation of Slug, Snail, Vimentin, ZEB1, MTA1, and MMP-9 (Pan et al., 2009; Li et al., 2013; Principe et al., 2017). Our results are consistent with the literature and show that PS significantly suppressed cell migration in HNC lines in a concentration-dependent manner.

Apoptosis represents a natural and programmed mechanism of cellular death that is a normal part of growth and development. An important anticancer effect of PS is that it facilitates the activation of major apoptotic signaling pathways (Hsiao et al., 2014; Chen et al., 2018; Liu et al., 2020). In the intrinsic pathway, the balance between Bax/Bcl-2 and the control of Casp-3 plays a critical role in the modulation of apoptotic signals (Safarzadeh et al., 2014). Therefore, proteins involved in apoptotic signaling pathways have been proposed as important molecular targets in cancer therapy (Xu et al., 2009). In the HeLa cell line, PS induced apoptosis by upregulating Casp-3 and −9 and downregulating Bcl-2 and Bcl-x (Tong et al., 2021). Similarly, PS treatment has been shown to promote a concentration-dependent increase in Casp-3 and Bax but decrease in Bcl-2 ratio in pancreatic, breast, prostate, and endometrial cancer cell lines (Chakraborty et al., 2010; Wen et al., 2017; Hsu et al., 2020). Additionally, PS treatment induced apoptosis in bladder (T24 and T24R) cancer cell lines by decreasing the expression of Bcl-2 and Bcl-xl, increasing Casp-3, and keeping Bax and Bad unchanged.^28^ In addition, PS treatment was observed to induce apoptosis in gastric cancer (AGS) cells by promoting an increase in the expression of Bad, Bax, cytochrome c, and Casp-3- and −9 and decreasing mitochondrial transmembrane potential (Liu et al., 2020). In addition, in a breast cancer (MDA-MB-468) xenograft mouse model study, PS was found to exhibit antiproliferative activity through concentration-dependent inhibition of Akt and upregulation of Bax (Wakimoto et al., 2017). Similarly, in a colon cancer (HT-29) xenograft mouse model, it was reported that PS caused a decrease in the expression of the antiapoptotic *Bcl-2* gene at the mRNA level and increased the expression of the proapoptotic *Bax*, *Bak*, *Bad*, and *Bid* genes (Priego et al., 2008). In the present study, the real-time PCR, western blotting, and immunofluorescence staining results show that PS in HNC cells in a concentration-dependent manner significantly induced apoptosis by increasing the expression of *CASP-3* and *BAX* gene mRNA, protein, and intracellular levels manner, while decreasing *BCL-2*. Moreover, the annexin-V/PI staining test demonstrated a concentration-dependent reduction in the number of apoptotic cells in PS-treated HNC cells. This was supported by the increased annexin-V positive cell population and the upregulation of key apoptosis-associated CASP-3 and BAX proteins, and the downregulation of BCL-2.

Apoptosis can induce inhibition of cell proliferation. PS (10–100 μM) was reported to induce apoptosis in melanoma (A375), prostate (MIA PaCa, PANC-1, MIA PaCa-2, MIA PaCa-2GEMR, LNCaP, and PC3), and lung cancer (A549) cell lines, with cell proliferation being inhibited concentration-dependently (Priego et al., 2008; Mena et al., 2012; Lin et al., 2012; Hsu et al., 2020). Additionally, treatment of cisplatin-resistant human oral cancer (CAR) cells with PS (50–75 μM) resulted in decreased cell viability through stimulation of apoptotic caspase activation and reduced Akt activation (Derici et al., 2021). Therefore, the inhibitory effect of PS on the proliferation of HNC cells may be associated with the induction of apoptosis.

HNC cell lines are well studied and defined in terms of genetic, epigenetic, and various marker expression with gene sequencing, isoenzyme analysis, band cytogenetics, PCR, cell classification, nucleic acid test, microscopic morphology control, growth curve analysis, colony formation, and STR analysis (DNA fingerprint) tests (Bamford et al., 2004; Göttgens et al., 2021). However, HNC cells produce some fibroblast-specific markers and proteins with a typical spindle-shaped fibroblast morphology (Rangan, 1972; Rheinwald and Beckett, 1981; Bamford et al., 2004; Ragin et al., 2004; Yin et al., 2024). Although HNC was modeled in vitro with the cell lines used in our study, it may not fully represent the heterogeneity of HNC due to variation in genetic, epigenetic, phenotypic, and functional properties (Göttgens et al., 2021; Martinez-Pacheco and O’Driscoll 2021; Van den Bossche et al., 2022). In the SCC-9 cell line, CDKN2A (homozygous) and TP53 (homozygous) genomic mutations have been reported (Bamford et al., 2004). Additionally, by sequencing for FaDu, CDKN2A (homozygous), SMAD4 (homozygous), TP53 (heterozygous), and TP53 (heterozygous) gene mutations have been reported (Bamford et al., 2004). There is no TP53 mutation in SCC-90 (Ragin et al., 2004). These variations may explain the differences in the response of HNC cell lines to PS treatment (Chehelgerdi et al., 2023). Additionally, cell–stroma and cell–microenvironment interactions may not be adequately mimicked in one-dimensional cell cultures (Fontana et al., 2021). Therefore, there is a need to validate our data with advanced preclinical studies involving three-dimensional culture, co-culture systems, organoids, animal models, or patient-derived tumor cells.

## 5. Conclusion

In the present study, the MTT assay confirmed the time- and concentration-dependent highly selective cytotoxic effect of PS on HNC cell lines with low IC_50_ values. The findings showed that PS can induce apoptosis in human HNC cells, thereby suppressing cell viability and proliferation. In addition, the cell migration assay revealed the potent inhibitory effect of PS on HNC cell migration. Moreover, the annexin-V-IP findings in flow cytometry confirmed the increase in apoptotic cell death in HNC cell lines in response to PS treatment. The real-time PCR, western blotting, and immunofluorescence staining results further confirmed that the underlying mechanism of PS’s antitumor activity is to induce upregulation of *CASP-3* and *BAX* and downregulation of *BCL-2* at mRNA, protein, and intracellular levels, associated with the activation of the apoptotic signaling pathway in HNC cells. The cytotoxic, antiproliferative, proapoptotic, and antimigratory effects of PS against HNC cells may be associated with its anticancer activity. The data from our study demonstrated for the first time that PS has anticancer activities in HNC cells. Therefore, the study has provided preliminary data for further research on the development of the clinical application potential of PS in the treatment of HNC in the future.

## Figures and Tables

**Table 1:** Characteristics of the cell lines.

ATCC No	Cell line	Tissue	Lesion type	Sex	Age
CCL-23	HEp-2	Larynx	Metastasis	Male	56
CRL-1629	SCC-9	Tongue	Primer	Male	25
CRL-3239	SCC-90	Tongue	Metastasis	Male	46
HTB-43	FaDu	Pharynx	Primer	Male	56
CCL-110	Detroit-551	Skin	Normal	Female	Fetus

ATCC: American Type Culture Collection.

**Table 2:** IC values of PS against human HNC and fibroblast cell lines.

Cell lines	IC_10_ (μM ± SD)		IC_30_ (μM ± SD)		IC_50_ (μM ± SD)		SI	
	24 h	48 h	24 h	48 h	24 h	48 h	24 h	48 h
HEp-2	9.5 ± 1.89	6.77 ± 1.43	48.39 ± 1.68	27.90 ± 1.86	87.54 ± 1.94	76.93 ± 1.72	3.12	3.26
SCC-90	4.3 ± 1.16	2.41 ± 1.25	54.01 ± 1.58	17.03 ± 1.44	76.09 ± 1.88	53.30 ± 1.72	3.5	4.71
SCC-9	6.8 ± 1.09	7.01 ± 1.11	58.01 ± 2.35	27.09 ± 2.04	95.56 ± 3.78	45.18 ± 4.12	2.85	5.56
FaDu	3.45 ± 0.34	1.99 ± 0.87	38.03 ± 2.01	15.98 ± 1.78	63.81 ± 2.96	30.11 ± 3.13	4.28	8.35
Detroit-551	121.3 ± 1.16	63.02 ± 1.45	157.08 ± 1.76	134.45 ± 1.59	273.2 ± 2.436	251.5 ± 2.40	-	-

HNC: Head and neck cancer cells. PS: Pterostilbene; IC: Maximum cell viability inhibition concentration; SD: Standard deviation; SI: Selectivity index.

* SI: (IC_50_ value for non-tumour cells)/IC_50_ for cancer cell.
